# Emerging CAR-Treg and regulatory T cell therapies for inflammatory bowel diseases: a systematic review of a new era of treatment

**DOI:** 10.3389/fimmu.2026.1920203

**Published:** 2026-09-03

**Authors:** Fabio Suarez-Trujillo, Theodore S. Steiner, Javier P. Gisbert, María Chaparro

**Affiliations:** 1 Inflammatory Bowel Disease Unit, Gastroenterology Department, Hospital Universitario de La Princesa, Instituto de Investigación Sanitaria Princesa (IIS-Princesa), Universidad Autónoma de Madrid (UAM), Centro de Investigación Biomédica en Red de Enfermedades Hepáticas y Digestivas (CIBEREHD), Madrid, Spain; 2 Department of Medicine, BC Children’s Hospital Research Institute, Division of Infectious Diseases, University of British Columbia, Vancouver, BC, Canada

**Keywords:** CAR-T, CAR-Treg, inflammatory bowel disease, regulatory T cells, regulatory T-cells therapy

## Abstract

**Background:**

Inflammatory bowel disease (IBD) is a chronic immune-mediated disorder characterized by dysregulated mucosal immunity. Despite major advances in therapies, a high proportion of patients do not achieve or sustain remission. Cellular immunotherapies have emerged as a mechanistically distinct therapeutic strategy with potential relevance for refractory disease. In the present paper, the authors aim to evaluate the rationale and current evidence supporting chimeric antigen receptor (CAR)-engineered immune cells and regulatory T (Treg)-cell–based therapies in IBD. Preclinical studies and early translational data support biological plausibility and feasibility of these therapies. However, available human evidence remains limited, with sparse clinical experience and short follow-up.

**Methods:**

A systematic review was conducted according to PRISMA guidelines. Search was performed in PubMed and EMBASE databases from inception to February 2026 including terms like “IBD”, “CAR-T”, “CAR-Treg” and “Treg therapy” among others. Eligible studies included preclinical, translational and clinical reports evaluating CAR-engineered immune cells in IBD models.

**Results:**

A total of 11 studies met the inclusion criteria after the screening and were included in the review. CAR-Treg technologies build upon advances achieved with conventional CAR-T cells in oncology and enable antigen-specific immune regulation rather than cytotoxicity. Preclinical studies and early translational data support biological plausibility and feasibility. However, available human evidence remains limited, with sparse clinical experience and short follow-up. Major unresolved issues include cellular persistence, phenotypic stability (particularly for Tregs), optimal antigen selection, manufacturing scalability, and long-term safety.

**Conclusions:**

Cellular immunotherapies represent a promising but still investigational therapeutic paradigm in IBD. Robust clinical trials with standardized endpoints and long-term safety monitoring are needed before their clinical adoption.

## 1 Introduction

Inflammatory bowel diseases (IBD) are a complex group of immune-mediated pathologies affecting the gastrointestinal tract from the upper portion to the anus. IBD are multifactorial diseases characterized mainly by a chronic inflammation of the gut epithelium and mucosa, and an exacerbated immune response (1, 2). Main forms of IBD include ulcerative colitis (UC) and Crohn’s disease (CD), which share immune and ethiopathogenic underlaying mechanisms, but differ in range of affection and clinical manifestations (1). Incidence of IBD is currently increasing worldwide, mostly affecting industrialized and high-income countries (3). In 2021, IBD accounted for an estimated 3.83 million prevalent cases and 375,140 incident cases worldwide, underscoring its growing global burden on healthcare systems (3, 4).

Causes of IBD are multifactorial and remain nowadays partially nuclear, although it is known that genetic background, environmental factors and dysbiosis clearly contribute to the local and peripheral immune system deregulation that leads to IBD onset and chronification (1, 2, 5). The loss of tolerance by the local immune system against luminal antigens, and gut epithelial barrier dysfunction give us the key clues for understanding the basis of IBD and enlighten the pathway for treatment; current drugs for IBD are focused on the immune system modulation, targeting upregulated proinflammatory pathways involved in the pathogenesis (6, 7).

In the last decade, biological and small-molecule based therapies have represented a significant advance in the treatment and clinical management of patients with IBD (8, 9). They are generally safe treatments with mechanisms of action that directly target the immune system by blocking and modulating some of the inflammatory pathways in UC and CD. Main biologic drugs include anti-TNFα (infliximab, adalimumab, golimumab), anti-integrin α4β7 (vedolizumab), anti-IL12/23p40 (ustekinumab) and anti-IL23p19 (risankizumab, mirikizumab, and guselkumab) mechanisms (7–13). Small molecules, in turn, target intracellular pathways implicated in the dysregulated inflammatory response in IBD, such as Janus kinases inhibitors (tofacitinib, filgotinib, upadacitinib) and the sphingosine-1-phosphate receptor modulator (ozanimod, etrasimod) (14, 15).

Despite the growing therapeutic armamentarium for IBD, only about one-third of patients achieve remission. In addition, among those who initially respond to biologic therapy, secondary loss of response is common, and only around 40% maintain remission after one year (9, 16, 17). Thus, despite the recent expansion of the therapeutic landscape in IBD, a considerable proportion of patients still fail to achieve adequate and sustained disease control. Moreover, biologic agents and small-molecule therapies rely on consistent adherence to maximize their effectiveness, which may itself become a limitation in real-world practice (3, 18). Consequently, IBD management remains a significant challenge for healthcare systems, highlighting the urgent need for novel therapeutic approaches that can improve treatment response and deliver sustained long-term benefit.

Chimeric antigen receptor T-cell (CAR-T) and regulatory T-cell (Treg)-based therapies are gaining interest as novel approaches for immune-mediated diseases, owing to their potential to selectively modulate pathogenic immune responses within the inflamed mucosal microenvironment and to promote inflammatory resolution with limited systemic immunosuppression (19–25). By directly targeting disease-relevant immune circuits, these approaches offer the potential for antigen-specific, mechanistically driven, and durable immune modulation, a paradigm particularly attractive for chronic inflammatory disorders characterized by immune dysregulation (26, 27).

Cell-based immunotherapies have already transformed the therapeutic landscape of oncology, demonstrating the feasibility and clinical impact of engineered T-cell approaches (28, 29). Building upon this success, similar strategies are increasingly being explored in immune-mediated diseases, where early clinical experiences suggest that targeted cellular therapies may restore immune homeostasis and induce sustained disease control (23–25).

These conceptual advances support the investigation of CAR-T and Treg-based therapies as next-generation therapeutic approaches for IBD management. Several therapeutic strategies aimed at restoring regulatory T-cell function are currently under investigation for IBD. These include adoptive transfer of ex vivo-expanded polyclonal Tregs, antigen-specific regulatory T cells, and pharmacological approaches designed to expand or enhance endogenous Treg populations through modulation of cytokine signaling, low-dose IL-2 administration, or tolerogenic immune pathways (20–23). Although these approaches have demonstrated encouraging preclinical and early clinical results, their efficacy may be limited by insufficient tissue specificity, variable persistence, and the potential for systemic immunosuppression (25–27). In this context, CAR-Treg therapy has emerged as a next-generation strategy that combines the suppressive capacity of regulatory T cells with antigen-specific recognition, enabling localized activation within inflamed tissues and potentially improving both efficacy and safety.

This systematic review aims to comprehensively evaluate and synthesize the current evidence regarding the application of CAR-T/CAR-Treg and Treg-based strategies in IBD. By integrating data across experimental and clinical studies, this work intends to provide a structured framework for understanding the therapeutic potential and future directions of Treg-based therapies, particularly CAR-Tregs, in IBD.

## 2 Methods

### 2.1 Literature search strategy

**Figure 1:**
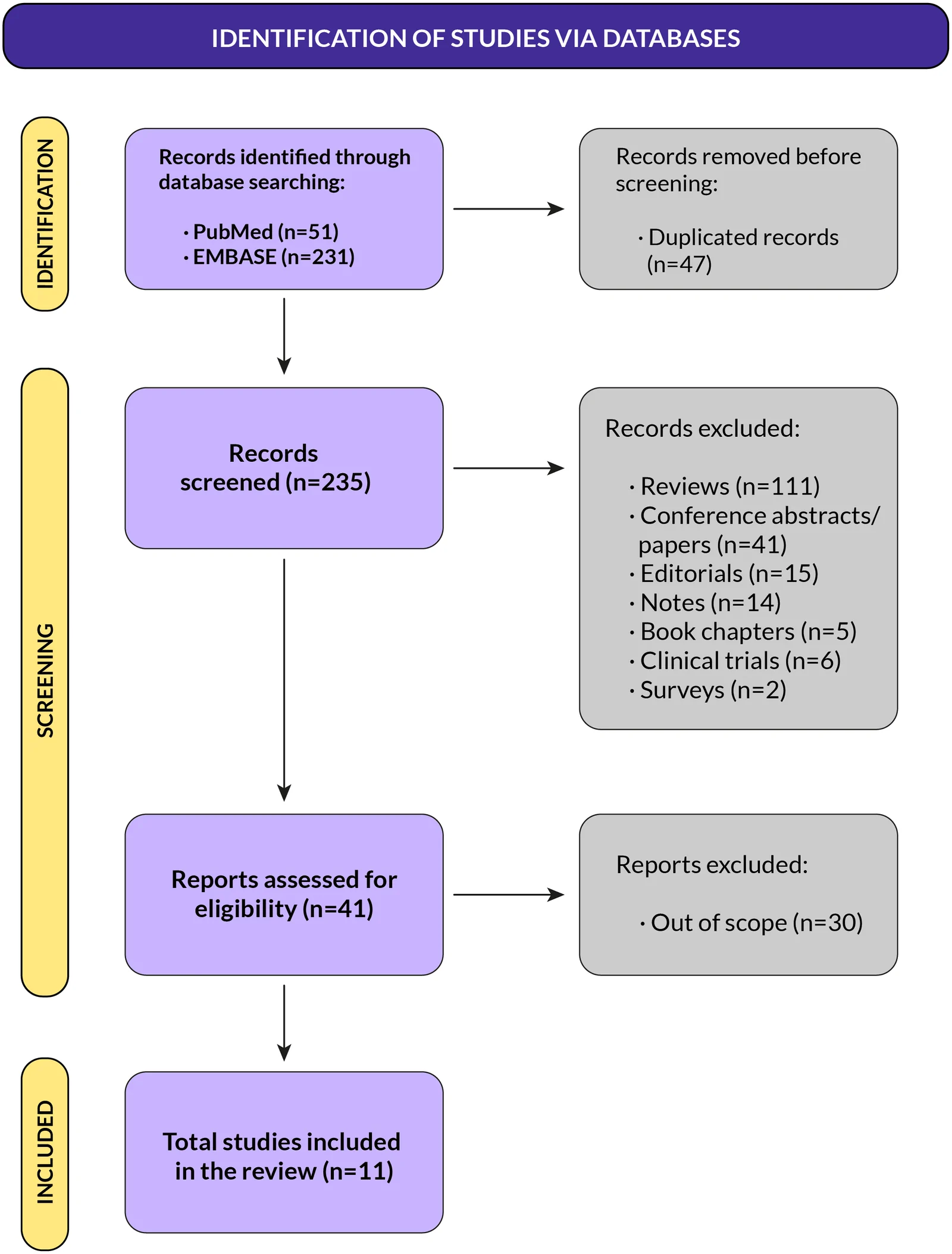
Flow chart detailing selection process of studies included in this review. Previous reviews, abstracts, notes, editorials, book chapters, active clinical trials and surveys were excluded in the screening process.

A systematic review of the scientific literature up to February 2026 was carried out within PubMed and EMBASE databases using the following algorithm: (“inflammatory bowel disease” OR “IBD” OR “ulcerative colitis” OR “Crohn’s disease”) AND (“chimeric antigen receptor” OR “CAR-T” OR “CAR-Treg” OR “CAR Treg” OR “antigen-specific regulatory T cells” OR “regulatory T cell therapy” OR “Treg therapy”). Study selection followed the PRISMA 2020 guidelines for systematic reviews (30). Narrative reviews, systematic reviews, editorials, and opinion articles were excluded. Only original articles, preclinical studies, clinical trials, and letters to the editor that provided experimental or clinical data were included, as shown in **Figure 1**.

## 3 Results and discussion

### 3.1 Emerging landscape and conceptual framework of cellular therapies in IBD

Cellular immunotherapies have emerged as a particularly attractive alternative, offering the possibility of mechanistically precise and durable immune modulation. Rather than broadly inhibiting inflammation, these approaches aim to reshape dysregulated immune networks that drive disease pathogenesis. Conceptually, Treg-based therapies, antigen-specific regulatory strategies, and CAR-engineered immune cells represent distinct but convergent efforts to restore immune tolerance. The early experimental and clinical studies included in this work suggest that cell-based interventions may exert effects beyond the specific cytokine pathways blockade achieved with biologics, potentially inducing long-lasting tolerogenic and regulatory states. However, the translational development of cellular therapies in IBD remains at an early stage, with major challenges involving safety, antigen selection, lineage stability, tissue trafficking, and scalable manufacturing.

In this review, a total of 11 research articles were retrieved following systematic database searches and manual eligibility assessment. These studies are discussed below, first addressing investigations performed in preclinical models of IBD, followed by studies conducted in human IBD context. **Tables 1**, **2** summarize the methodological characteristics and principal findings of the included preclinical and translational Treg and CAR-T/CAR-Treg studies, respectively.

**Table 1:** Summary of studies included in the review evaluating preclinical and translational regulatory T cell approaches in inflammatory bowel disease.

Reference	Approach	Therapeutic strategy	Target	Sample size	Main findings
Khan et al., 2025 (55)	Animal model (DSS induced colitis in humanized mice)	Autologous regulatory T cells	–	n=4 per group	Autologous Treg therapy effectively ameliorated the symptoms of DSS-induced colitis in a humanized mouse model by reducing the DAI by 78% and weight loss, and also preserving colonic length and improving stool consistency and intestinal bleeding scores compared to vehicle-treated groups. Treg therapy also reduced histological inflammation and crypt damage induced by DSS.
Meng et al., 2025 (57)	Animal model (DSS induced colitis)	Bone marrow niche-resident regulatory T cells transference	–	n=7–20 per group	This study robustly demonstrates that the transfer of bone marrow niche-residential Tregs effectively ameliorates clinical and histopathological symptoms of DSS induced colitis, reduce inflammation by inhibiting Th17 cells, and crucially promote colon tissue regeneration by preferentially homing to crypt bases. The key mechanism underlying these effects is mediated by the ectoenzyme CD39 expressed by bone marrow niche Tregs.
Cheng et al., 2025 (58)	Animal model (different T cell adoptive transference models)	Polyclonal regulatory T cell tranference	–	n=3 per group	This study highlights one side effect of Treg therapy for treating different immune-mediated disorders (including IBD): transferred Tregs drive the expansion of IL-17 producing Th17 pathogenic cells by reducing IL-2 and relying on IL-6 and TGF-β signaling. This adverse effect can be mitigated with anti-IL-6 antibodies or by blocking the IL-6/STAT3 pathway, which significantly improves the therapeutic efficacy of Treg cell therapy in treating colitis.
Desreumaux et al., 2012 (59)	Study involving humans	Antigen-specific Tregs adoptive therapy	Ovalbumin	n=20	Antigen-specific Treg therapy using ova-Tregs is well-tolerated in patients with refractory CD, showing significant improvements in CDAI and IBDQ scores, and a correlation between reduced ova-specific immune responses and clinical benefit. Ova-specific proliferative responses of PBMCs from responder patients are significantly lower than those from non-responders, what correlates with clinical benefit and followed the dynamics of CDAI reduction.
Canavan et al. 2014 (61)	Study involving humans	Tregs adoptive therapy	–	n=13 (blood donors for Treg isolation)	*In vitro* expanded CD45RA+ Tregs derived from CD patients not only demonstrate robust suppressive capabilities and phenotypic stability, resisting conversion to pro-inflammatory phenotypes, but also possess the necessary homing receptors to target inflamed gut tissue.
Voskens et al. 2017 (60)	Study involving humans	Autologous Treg therapy	–	n=8 (UC blood donor patients)	The GMP-compliant protocol developed for the *ex vivo* expansion of stable, functional, and polyclonal CD25+ Tregs from patients with UC exhibit strong immunosuppressive capabilities and express gut-homing markers in both CD4+ and CD8+ T cells, making them suitable candidates for a phase I clinical trial to assess their safety and tolerability in UC patients.
Voskens et al., 2022 (62)	Study involving humans	Autologous Treg therapy	–	n=1	Adoptive transfer of autologous, *ex vivo* expanded polyclonal Tregs can induce clinical, biochemical, endoscopic, and histological responses in refractory UC. The observed increase in mucosal Tregs and upregulation of IL-10 supports the mechanism that transferred Tregs migrate to the inflamed colon and ameliorate local disease activity. Infunded Tregs have also a transient but marked biochemical response in associated PSC, evidenced by the improvement in liver enzyme levels.

CD, Crohn’s disease; CD4, cluster of differentiation 4; CD8, cluster of differentiation 8; CD25, cluster of differentiation 25; CD39, cluster of differentiation 39; CD45, cluster of differentiation 45; CDAI, Crohn’s disease activity index; DAI, disease activity index; DSS, dextran sodium sulphate; IL, interleukin; GMP, good manufacturing practices; IBD, inflammatory bowel disease; IBDQ, inflammatory bowel disease questionnaire; Ova, ovalbumin; PSG, primary sclerosing cholangitis; STAT3, signal transducer and activator of transcription 3; TGFβ, transforming growth factor-beta; Treg, regulatory T cell; UC, ulcerative colitis.

**Table 2:** Summary of studies included in the review evaluating preclinical clinical chimeric antigen receptor-T cell and regulatory chimeric antigen receptor-T cell approaches in IBD.

Reference	Approach	Therapeutic strategy	Target	Sample size	Findings
Blat et al., 2014 (83)	Animal models (adoptive transference of CEA specific CAR effector CD4+ cells and azoxymathane-DSS induced colitis)	CAR-Treg	Carcinoembryogenic antigen (CEA)	n=7 per group	This sudy demonstrate that CEA-specific CAR Tregs significantly suppressed the severity of adoptive transference colitis, preventing weight loss and reducing colitis scores compared to control Tregs or no treatment. They also showed a significant colitis-suppressive effect in a azoxymethane-DSS model (colitis and associated colon cancer model), even in innate-immunity-mediated stages of the disease, and significantly decreased the subsequent colorectal tumor burden.
Boardman et al., 2022 (86)	Animal model (TNBS induced colitis)	CAR-Treg	Bacterial flagellin (FliC)	n=7 per group	This study provides strong evidence for the therapeutic potential of flagellin-specific CAR-Tregs in treating IBD. Authors demonstrate specific activation, potent immunosuppression, preferential homing to inflamed intestinal tissues, and the ability to promote epithelial barrier integrity, making them a promising candidate for further clinical investigation in IBD therapy.
Cui et al., 2025 (90)	Animal model (humanized mice model for IBD)	CAR-Treg	IL-23 receptor (IL-23R)	–	Engineered IL23R-CAR Tregs maintain their regulatory function and exhibit antigen-specific immunosuppressive fuction both *in vitro* and *in vivo*. They also show ability to migrate to IL23R-expressing tissues and their specific activation by CD patient-derived cells. In a xenograft mouse model, IL23R-CAR Tregs migrated specifically to IL23R-positive tumors, but not to IL23R-negative tumors, demonstrating their capacity to home to tissues expressing their target antigen.
Müller et al., 2025 (91)	Study involving humans	CAR-T	CD19	n=1	CD19 CAR T-cell therapy can induce remission in a patient with severe multidrug-resistant ulcerative colitis. The patient achieved clinical and biochemical remission, which was sustained over a 14-week follow-up period without the need for concomitant therapy. Endoscopic, histologic, and ultrasonographic assessments indicated signs of mucosal healing over time.

CAR, chimeric antigen receptor; CAR-Treg, chimeric antigen receptor regulatory T cell; CEA, carcinoembryogenic antigen; CD, Crohn’s disease; CD4, cluster of differentiation 4; CD19, cluster of differentiation 19; DSS, dextran sodium sulphate; IBD, inflammatory bowel disease; IL-23R, interleukin 23 receptor; TNBS, 2,4,6-trinitrobenzene sulphonic acid.

### 3.2 Polyclonal Treg adoptive therapies

Treg cells are the key players in the peripheral and central immune tolerance and are also essential for maintaining intestinal immune homeostasis. The conventional Treg population derived from the thymus is defined by expression of the transcription factor FOXP3 and, in humans, they can also be functionally characterized by surface antigens as CD4+CD25^hi^CD127^lo/-^ (31, 32). Functionally, FOXP3^+^ Tregs suppress immune responses through multiple mechanisms, including inhibitory receptor signaling and secretion of regulatory cytokines. Hallmark immunosuppressive mediators released by Tregs include IL-10, IL-35 and TGFβ, which contribute to the attenuation of effector T-cell function and induce and maintain local tolerance (33, 34). Furthermore, Tregs are also able to mediate the suppression of pro-inflammatory environment by expressing contact-dependant inhibitory signals such as CTLA-4 and ICOS, and by capturing high quantities of IL-2 (by expressing CD25) (35–37). Treg cells also interact with the innate immunity by inhibiting dendritic cell maturation (downregulating the expression of CD80 and CD86), dampening pro-inflammatory cytokine production, and promoting regulatory phenotypes in innate immune cells (35). In addition to thymus-derived FOXP3^+^ Tregs, distinct regulatory T-cell subsets lacking constitutive FOXP3 expression have been described. Among these, type 1 regulatory T (Tr1) cells represent a population primarily characterized by high production of IL-10 and suppressive activity, often associated with markers such as LAG-3 and CD49b (38, 39).

The intestinal Treg compartment exhibits substantial heterogeneity, including a specialized subset of gut-resident Tregs co-expressing FOXP3 and RORγt, which is closely linked to microbiota-driven immune adaptation (40, 41). Experimental studies indicate that approximately 20-30% of colonic lamina propria FOXP3^+^ Tregs express RORγt, highlighting their relevance in mucosal tolerance (42). They are involved in the short-chain fatty acids metabolism and express strong immunoregulatory markers such as CD39, CD44, CD73, CTLA-4, IRF4, ICOS and IL-10 (40). Beyond their well-established immunosuppressive activity, accumulating evidence indicates that Tregs actively contribute to intestinal tissue repair and epithelial regeneration (43–45). Tissue-resident Tregs promote mucosal healing through the production of amphiregulin, a member of the epidermal growth factor family that supports epithelial proliferation, barrier restoration, and recovery following inflammatory injury (43–45). In addition, Tregs interact closely with epithelial cells, stromal cells, and innate immune populations to coordinate tissue remodeling and resolution of inflammation. These reparative functions are particularly relevant in IBD, where sustained restoration of epithelial integrity is increasingly recognized as a critical therapeutic objective alongside immune suppression (43–47). Consequently, successful Treg-based therapies may provide clinical benefit not only by limiting pathogenic immune responses but also by actively promoting mucosal repair and long-term tissue homeostasis. Dysregulation of regulatory T-cell networks contributes to IBD pathogenesis, providing the rationale for Treg-based cellular therapies aimed at restoring immune balance.

Clinical experience with polyclonal Tregs in immune-mediated diseases is still limited but has provided important proof-of-concept evidence. Studies in type 1 diabetes, have shown that infusion of ex vivo–expanded autologous Tregs was well tolerated and that transferred cells could persist in circulation for up to one year, with indications of preserved β-cell function (48, 49). Similarly, adoptive Treg transfer has been also tested in systemic lupus erythematosus (SLE), demonstrating transient persistence and signs of local immunological activity in inflamed tissues (50). Additional proof-of-concept data come from graft-versus-host disease (GvHD), where early clinical studies demonstrated the feasibility, safety, and *in vivo* persistence of adoptively transferred Tregs. Initial trials showed clinical improvement following infusion of expanded CD4^+^CD25^+^CD127^−^ Tregs (51), while later studies reported reduced incidence of acute GvHD after infusion of cord blood–derived Tregs in transplant recipients (52). Together, these findings support the safety and potential immunomodulatory activity of polyclonal Treg therapy in human inflammatory conditions.

Regarding regulatory Treg–based therapies in IBD, the available clinical evidence is limited to early-phase clinical studies and case reports. The studies identified in this review (**Table 1**) evaluated the feasibility and safety of adoptive transfer of ex vivo–expanded autologous Tregs obtained from peripheral blood (CD4^+^CD25^+^CD127^−^) and are primarily focused on patients with severe or treatment-refractory disease. Main outcomes of these studies are clinical activity indexes, endoscopic findings, inflammatory biomarkers, and persistence of transferred cells in peripheral blood. Some of the studies also focused on the ex vivo expansion of stable Treg subsets from patients with IBD as a preparatory step for potential adoptive cell therapy. The available clinical literature on this regard remains limited in size and heterogeneity, with most studies designed to evaluate feasibility, safety, and preliminary immunological effects rather than therapeutic efficacy. Ongoing clinical trials such as TRIBUTE and ER-TREG01 reflect the growing interest in adoptive regulatory T-cell therapies for IBD and are expected to further advance the clinical development of these cellular therapeutic strategies, although only protocols and not clinical outcomes have been published up to the time of this review (53, 54).

#### 3.2.1 Treg approaches tested in animal models

Khan et al. established a peripheral blood mononuclear cells (PBMCs)-humanized NOG mouse model to evaluate autologous Treg therapy in DSS-induced colitis, addressing a key translational limitation of conventional murine models for testing human cell therapies (55). In each experimental cohort, PBMCs from a single healthy donor were used both to reconstitute the human immune compartment and to isolate/expand Tregs for treatment, minimizing allogeneic confounding and inter-donor variability. The authors verified the quality of the expanded product by flow cytometry. Therapeutically, autologous Treg infusion improved clinical outcomes compared with vehicle and comparator cell controls, reducing disease activity scores and improving stool consistency/bleeding measures (55). The study further positions this model as a practical preclinical bridge to support human-specific immunotherapy testing, particularly relevant for interventions that cannot be meaningfully assessed in immunocompetent mice. This work provides a translationally oriented proof-of-concept that adoptively transferred, donor-matched human Tregs can ameliorate intestinal inflammation in a humanized inflammatory model.

Meng et al. studied if bone marrow niche-resident Tregs—a tissue-adapted regulatory population previously implicated in immune privilege— can suppress experimental colitis more efficiently than conventional lymphoid or non-niche Tregs (56). The authors compare transfers of CD150^hi^ bone marrow niche Tregs versus control Treg populations (CD150low and lymph-nodes Tregs) and assess clinical and histopathological outcomes (57). A major mechanistic emphasis is placed on CD39, which is highly expressed by niche Tregs and links their suppressive function to purinergic/adenosine-mediated immunoregulation. Functionally, niche Treg transfer reduced inflammatory Th17 responses in the colon, including Th17 fractions with high IL-17 and TNF-α expression, whereas control Treg populations did not achieve the same effect. Importantly, targeted deletion of CD39 in transferred Tregs largely depleted the therapeutic benefit, supporting CD39 as a key functional determinant of this regulatory function (57). These data suggest that tissue-specialized Treg subsets may provide high therapeutic potency, which may be of relevance for IBD, where efficient mucosal regulation and tissue repair must occur within a complex inflammatory microenvironment.

In contrast to efficacy-focused reports, Cheng et al. provide a rigorous mechanistic analysis showing that polyclonal adoptive Treg therapy can paradoxically enhance Th17 local response in an IBD model. Using a Rag1^−/−^ T cell transfer system (CD4+ CD45RB^hi^ effector T cells ± Tregs), the authors show that the presence of transferred Tregs suppresses Th1/Th2-type outputs but selectively increases Th17 differentiation from effector T cells in lymphoid and colonic tissues (58). Mechanistically, these authors demonstrated that Tregs elevate the pSTAT3/pSTAT5 balance in effector cells by suppressing IL-2 availability in the environment (via reduced IL-2 secretion and competitive consumption), thereby favoring IL-6/TGF-β-dependent Th17 polarization. A particularly important translational implication is that IL-2 deficiency not only increases Th17 differentiation but may also imprint a more pathogenic transcriptional profile, with upregulation of inflammatory pathways (e.g., IL23R-associated axis). Notably, the authors propose a mitigation strategy: blockage of the IL-6/STAT3 axis reverses Treg-induced pathogenic Th17 generation and improves the net therapeutic effect of adoptive Treg transfer (58). This study is therefore a key reminder that Treg therapies are not uniformly anti-inflammatory and that outcome is highly contingent on the cytokine landscape, dosing/context, and combinatorial approaches.

#### 3.2.2 Clinical Treg-based approaches in IBD

The Crohn’s And Treg Cells Study (CATS1) conducted by Desreumaux et al. tested a single infusion of autologous ovalbumin-specific type 1 regulatory T cells (ova-Tr1) in 20 patients with refractory CD in an open-label, multicenter, dose-escalation phase 1/2a design (59). The trial primarily assessed safety/tolerability and explored clinical and biological signals, using the Crohn’s disease activity index or the inflammatory bowel disease questionnaire and inflammatory markers alongside immunomonitoring of PBMC proliferative responses to ovalbumin. Overall, ova-Tr1 were well tolerated (54 adverse events; only 2 related to the test reagent) with 11 serious adverse events (3 related, all recovered). A clinical response was observed in 40% of patients at weeks 5 and 8, with an apparent dose-related signal: in the 10^6^-cell infusion cohort, 75% responded at weeks 5 and 8, and remission occurred in 38% at week 5 and 25% at week 8. Ova-specific proliferative responses of PBMCs from responder patients were significantly lower than those from non-responders at week 8, and the effect on proliferation was specific to ovalbumin, as no difference was found in PBMC proliferative responses to other antigens like tetanus toxoid or purified protein derivative. This reduction in ovalbumin-specific proliferation correlated with clinical benefit and followed the CDAI reduction. These findings support the immune-suppressive mechanisms of ova-Tregs and highlight the potential of this immune therapy approach, warranting further clinical and mechanistic studies for refractory CD (59).

Voskens et al. report a single-patient experience of adoptive transfer of autologous, ex vivo expanded polyclonal Tregs in refractory ulcerative colitis with concomitant primary sclerosing cholangitis (60). The patient received 1×10^6^ Tregs/kg and experienced a gradual improvement in symptoms, including a reduction in stool frequency and the absence of rectal bleeding. The clinical disease activity score (partial Mayo Score) decreased significantly from 7 points at baseline to 4 points by week 2 and 2 points by week 8 after Treg transfer. There was also a significant and rapid reduction in fecal calprotectin levels, accompanied by mucosal enrichment of CD3^+^/FOXP3^+^ and CD3^+^/IL-10^+^ T cells, and increased mucosal TGF-β and amphiregulin, a key mediator of Treg-driven epithelial repair and mucosal regeneration, supporting the concept that adoptively transferred Tregs may promote tissue healing in addition to immune regulation (60). The observed increase in mucosal Tregs and upregulation of IL-10 supports the mechanism that transferred Tregs migrate to the inflamed colon and ameliorate local disease activity, providing a strong rationale for further clinical trials of adoptive Treg therapy in refractory UC. The report also notes a marked but transient improvement in primary sclerosing cholangitis-associated liver enzymes, with the strongest effect lasting ~4 weeks before trends began to reverse, underscoring that durability may be limited after a single infusion of Tregs (60).

Canavan et al. provide an important translational framework for autologous Treg therapy in Crohn’s disease by comparing expansion of CD4^+^CD25^+^CD127^lo^CD45RA^+^ vs CD45RA^−^ Treg subsets under a workflow designed to be transferable to good manufacturing practices (GMP) (61). They show feasibility to expand CD patient-derived Tregs to a potential target dose within ~22–24 days, and demonstrate that CD45RA^+^-derived expanded Tregs maintain an epigenetically stable FOXP3 locus and are more resistant to undergo Th17 conversion than CD45RA^−^-derived lines. Beyond stability, the study addresses practical barriers to IBD translation: expanded CD45RA^+^ Tregs expressed homing-associated molecules (including α4β7, CD62L, CCR7) and demonstrated trafficking to human small bowel in a xenotransplant model, while also suppressing activation of lamina propria and mesenteric lymph node lymphocytes derived from inflamed CD mucosa (61). These results position CD45RA^+^ precursors as a rational starting population for clinical-grade autologous products in CD, specifically to mitigate the risk of inflammatory plasticity in a cytokine-rich intestinal environment.

Voskens et al. focus on a good manufacturing practices-conform expansion protocol for UC patient-derived CD25^+^ cells using IL-2, rapamycin, and anti-CD3/anti-CD28 beads, generating a polyclonal product designed as a backbone for early-phase clinical translation (62). The expanded cells were hypomethylated at intron 1 of the *Foxp3* locus (supporting lineage stability) and displayed suppressive capacity against both autologous peripheral blood responder cells and allogeneic colon-derived responder cells in carboxyfluorescein diacetate succinimidyl ester (CFSE)-based assays. Mechanistically, the authors report that function was mediated by soluble factors, including toxic granules, and they also observed induction of suppressive CD8^+^ subsets during expansion, which is relevant from a manufacturing-control standpoint (product definition/purity and release criteria) (62). Overall, this study does not constitute a therapeutic efficacy study per se, but it provides a detailed, disease-relevant manufacturing and characterization pipeline that directly supports the feasibility of clinical dosing studies in UC.

While these early observations support the conceptual promise of Treg-based interventions, a deeper evaluation of their therapeutic potential is needed and will require rigorously controlled clinical trials integrating mechanistic and clinical endpoints; critical questions regarding optimal cell sources, dosing strategies, *in vivo* persistence, and functional stability within inflammatory environments of Treg therapies remain unresolved.

### 3.3 CAR-T and CAR-Treg based therapies

**Figure 2:**
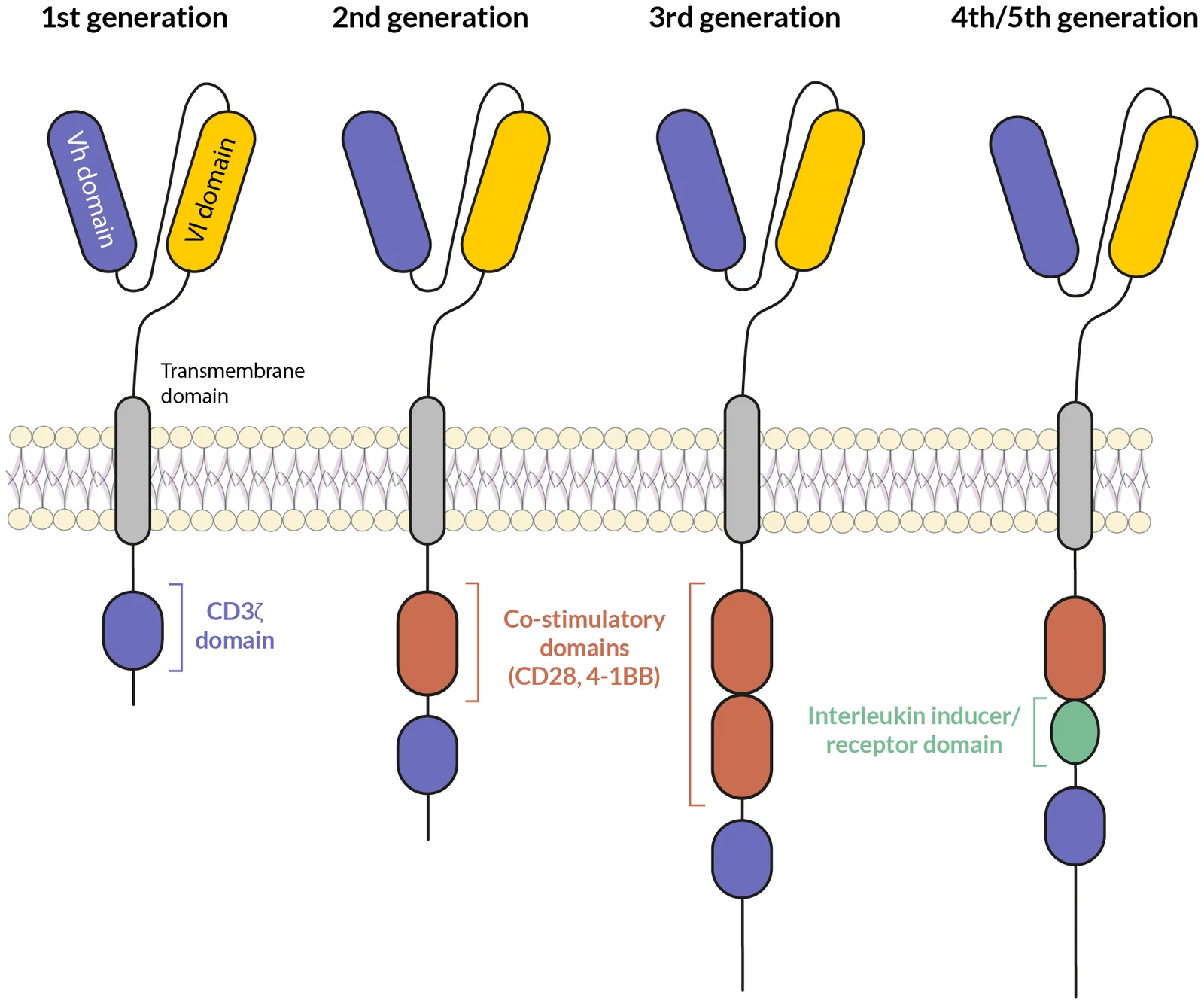
Structure evolution of CAR generations. First-generation CARs are structurally composed of an extracellular single-chain variable fragment (scFv), which in turn consists of the variable light (Vl) and heavy (Vh) chains of an antibody previously validated for specific target recognition, joined by a peptide linker. This structure is fused to an intracellular CD3ζ domain responsible for signal transduction. Second- and third-generation CARs share this primary core architecture; however, their intracellular domains have been modified to incorporate co-stimulatory signaling domains, most commonly CD28 and/or 4-1BB. Fourth-generation CARs additionally include an interleukin expression module (i.e.: IL-12), whereas fifth-generation CARs are engineered with an intracellular cytokine receptor domain (i.e.: IL-2Rβ), typically featuring STAT3/5 binding capability.

CAR therapy is an advanced form of adoptive cellular immunotherapy based in genetically engineered T lymphocytes that express an artificial surface receptor able to selectively recognize a target antigen in a major histocompatibility complex (MHC)-I independent manner (63, 64). The CAR construct itself is mainly composed of an antibody-derived extracellular recognition domain (single-chain variable fragment, scFv), intracellular signaling domains, and co-stimulatory molecules needed for the complete activation and function of the genetically engineered cell (62–66). The main region of this construct, the scFv domain, is derived from the variable (heavy, Vh, and light, Vl) regions of an antibody which is previously hybridoma-produced and tested for binding the target molecule. Successive generations of CAR designs have incorporated co-stimulatory domains such as CD28 or 4-1BB, which significantly enhance T-cell proliferation, persistence, and functional activity (67–69) (**Figure 2**). Intracellular and co-stimulatory domains can be switched for obtaining the most effective combination for the CAR-T cell activation and effect. The expression of this construct in the host cell is mediated by the latest-generation viral vectors, with which previously isolated effector T cells are transfected and then *in-vitro* stimulated and expanded for reinfusion (70, 71).

**Figure 3:**
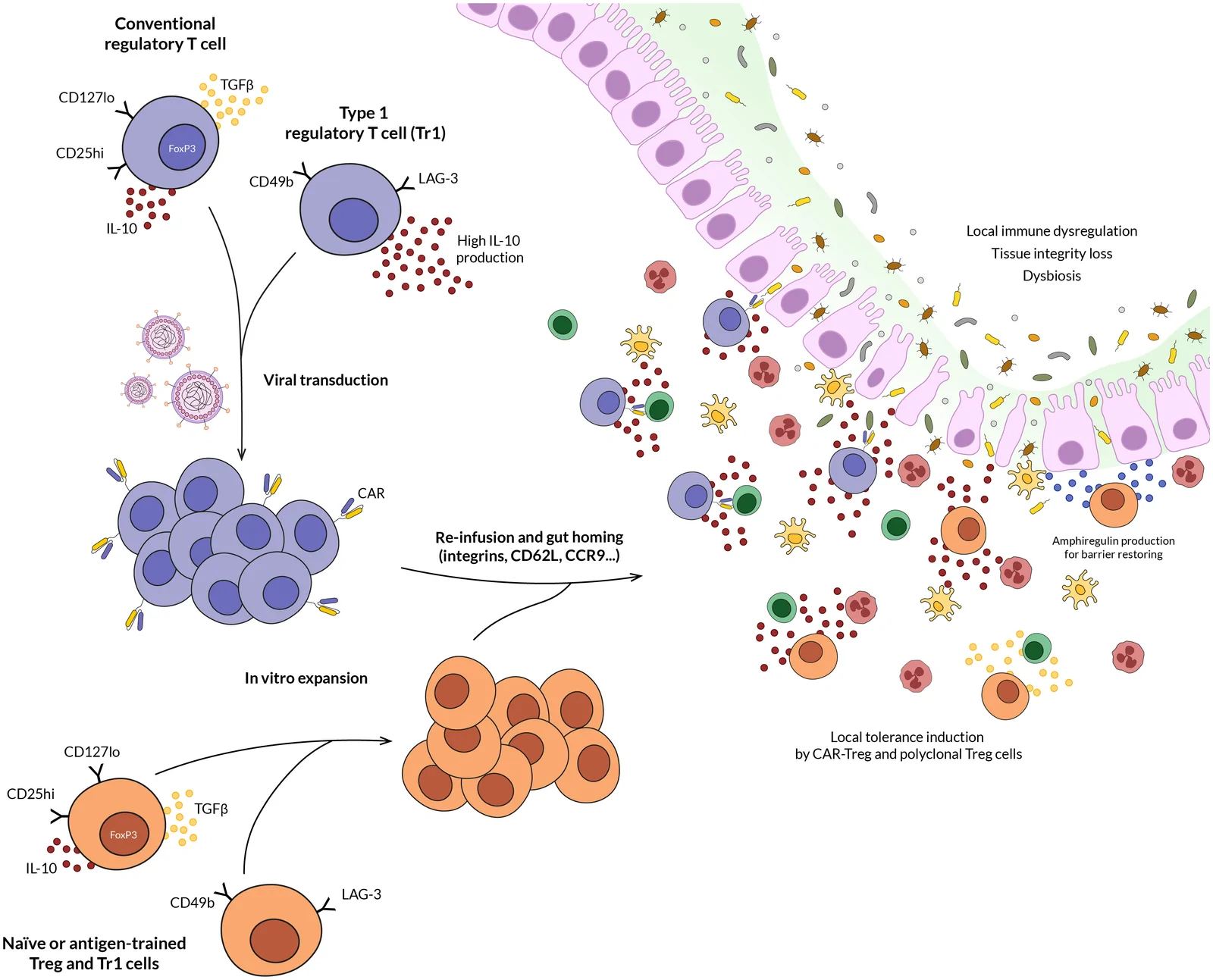
Generation and proposed mechanism of action of CAR-Tregs and polyclonal Treg therapies for IBD. Conventional regulatory T cells (CD4^+^CD25^hi^CD127^lo^FOXP3^+^) or type 1 regulatory T (Tr1) cells (CD49b^+^LAG3^+^FOXP3^−^) are isolated from peripheral blood. For CAR-Treg therapies, these cells are genetically engineered by viral transduction to express a chimeric antigen receptor (CAR) directed against an IBD-associated target antigen. In polyclonal approaches, Tregs or Tr1 cells are isolated and may be expanded in the presence or absence of a specific antigen. Following ex vivo expansion with IL-2, CAR-Tregs or polyclonal Tregs are reinfused into the patient, where they traffic to the inflamed intestinal mucosa through the expression of gut-homing molecules such as integrin α4β7, CD62L, and CCR9. Upon antigen recognition, CAR-Tregs undergo antigen-dependent activation, whereas polyclonal Tregs are activated through their native T-cell receptor. Activated regulatory cells exert localized immunomodulatory effects through the release of anti-inflammatory mediators, including IL-10 and TGF-β, thereby suppressing pathogenic immune responses and promoting local immune tolerance. In addition, they contribute to epithelial barrier restoration and mucosal healing through the production of tissue-reparative mediators such as amphiregulin, ultimately promoting the restoration of intestinal immune homeostasis.

CAR-Treg technology is a variant of CAR-T therapy toward immune regulation rather than cytotoxicity (23–25). In this framework, CAR constructs are introduced into Treg populations to confer antigen-dependent activation while preserving their intrinsic suppressive function. Unlike conventional CAR-T approaches designed for target cell elimination, CAR-Tregs exploit regulatory lymphocyte subsets as vehicles for localized immune modulation. This strategy may involve both FOXP3-expressing Tregs and Tr1 cells, depending on the intended functional and translational context (23–25, 72). By coupling regulatory cell activity to defined antigenic targets, CAR-Treg platforms aim to achieve spatially restricted immunoregulation, selectively attenuating pathogenic immune responses within diseased tissues. The modular nature of CAR constructs further allows tuning of activation thresholds and functional outputs, providing opportunities to tailor regulatory responses according to disease-specific requirements. In chronic inflammatory disorders such as IBD, where immune dysregulation is compartmentalized within the intestinal mucosa, CAR-Treg strategies offer a mechanistically distinct approach to restore immune homeostasis without relying on systemic immunosuppression (**Figure 3**).

The clinical success of CAR-T cell therapies has transformed the therapeutic landscape on hematologic malignancies, leading to multiple clinical trials and approvals for relapsed or refractory B-cell cancers (73). CD19-directed CAR-T products, including tisagenlecleucel, axicabtagene ciloleucel, and lisocabtagene maraleucel, have demonstrated remarkable efficacy in acute lymphoblastic leukemia, diffuse large B-cell lymphoma, and other B-cell neoplasms, establishing engineered cellular immunotherapy as a clinically validated modality (73–76). Beyond oncology, the deep immune remodeling induced by CAR-T-mediated lymphocyte depletion has stimulated growing interest in extending this approach to immune-mediated diseases. Recent exploratory studies and early clinical reports suggest that CAR-T approaches may achieve therapeutic benefit in immune-mediated diseases characterized by pathogenic immune cell populations, thereby supporting the concept of immune resetting through targeted cellular interventions. Initial clinical studies have demonstrated encouraging results in patients with severe systemic lupus erythematosus refractory to conventional therapies, where CD19 CAR-T cell therapy induced rapid clinical remission accompanied by disappearance of autoantibodies and sustained B-cell depletion (77, 78). Similar therapeutic responses have subsequently been reported in other antibody-mediated autoimmune conditions, including idiopathic inflammatory myopathies (79) systemic sclerosis (80), antiphospholipid syndrome (81) or autoimmune hemolytic anemia (82), suggesting that CAR-T-mediated B-cell depletion may represent a promising strategy for diseases characterized by autoreactive B-cell activity.

The available clinical data regarding CAR-T or CAR-Treg therapies in IBD primarily involve the use of CD19-targeted CAR-T cells, reflecting strategies aimed at depleting B-cell populations implicated in immune dysregulation. Only preclinical works have been published regarding CAR-engineered Tregs as a therapy for IBD, including the development of cells directed against intestinal antigens, microbiota-associated targets, and molecules involved in key inflammatory pathways. These studies have typically employed murine models of colitis to evaluate the capacity of engineered cells to localize to inflamed tissue and modulate immune responses. Recent translational efforts have focused on the generation and optimization of antigen-specific CAR-Tregs, including the selection of appropriate target antigens and strategies to enhance *in vivo* stability and suppressive function (**Table 2**).

#### 3.3.1 CAR-T and CAR-Treg approaches tested in animal models of IBD

Among the studies included in the present review, only three works provide mechanistic proof-of-concept for CAR-engineered regulatory strategies in preclinical models of IBD (**Table 2**). These works explore distinct targeting paradigms, focusing either on host-derived inflammation-associated antigens or microbial structures relevant to mucosal immune activation.

Blat et al. developed carcinoembryonic antigen (CEA)-specific CAR-modified regulatory T cells and evaluated their function in complementary murine models of colitis (DSS) and colitis-associated cancer (AOM-DSS) (83). Using CEABAC transgenic mice to ensure antigen availability, the authors demonstrated preferential accumulation of CEA-specific CAR-Tregs within the colon, accompanied by attenuation of inflammatory activity. Importantly, CAR-Treg transfer reduced disease severity and limited tumor burden in the AOM–DSS model, suggesting that antigen-restricted immune regulation may exert both anti-inflammatory and tissue-protective effects. However, rapid loss of detectable CAR-Tregs following infusion highlighted potential limitations in local persistence, a parameter of particular relevance for translational considerations of this kind of therapies (83). Published in 2014, this study provides early evidence that CAR engineering can re-direct regulatory T-cell activity to inflamed tissues, supporting the hypothesis that antigen-specific regulatory approaches may outperform polyclonal approaches. These findings are consistent with earlier demonstrations that redirected regulatory T cells with defined antigen specificity effectively suppress experimental colitis, reinforcing the biological plausibility of targeted immune modulation strategies (84, 85).

Boardman et al. engineered human naïve Tregs to express a CAR specific for E. coli H18 flagellin (FliC), aiming to couple suppressive function to a microbial antigen relevant to intestinal inflammation (86). After isolation, transduction and expansion, anti-FliC CAR-Tregs displayed successful antigen-dependent activation and enhanced suppressive function *in vitro*. Authors also tested the FliC-CAR-Treg function in human-derived monolayer colonoids assays and observed that CAR-Tregs improved barrier-associated readouts in terms of trans-epithelial electrical resistance (TEER), which is consistent with the protective function of this approach within the epithelial compartment homeostasis. For *in vivo* testing, FliC-CAR-Treg were infunded into humanized NSG mice to which TNBS was given for colitis development, revealing preferential intestinal localization and activation signatures associated with CAR expression. However, substantial background activation and model-specific constraints precluded definitive evaluation of therapeutic efficacy, highlighting persistent experimental challenges (86). Conceptually, targeting microbial antigens is supported by extensive evidence identifying flagellins as immunodominant antigens in CD (87–89), although inter-individual variability in microbial antigen exposure remains a critical translational consideration.

Cui et al. developed an antigen-specific regulatory strategy based on IL23R-targeted CAR-engineered Tregs, aiming to selectively modulate the IL-23/Th17 pathway central to CD pathogenesis (90). Human Tregs were genetically modified using lentiviral vectors encoding an IL23R-specific CAR, followed by ex vivo expansion and phenotypic validation to ensure preservation of canonical regulatory markers. IL23R-CAR-Tregs exhibited antigen-dependent activation and retained suppressive capacity, supporting the feasibility of redirecting regulatory function toward cytokine receptor targets. The CAR was evaluated in a xenograft tumor mouse model for *in vivo* trafficking and IL-23R binding testing of the construct. Experiments demonstrated attenuation of the inflammation following CAR-Treg transfer. Treated animals showed reduced disease severity and modulation of inflammatory immune signatures, consistent with selective interference in IL-23-driven pathogenic circuits (90). Mechanistically, the study supports the concept that CAR-Tregs may act not only through cell-cell suppression but also through targeted regulation of disease-relevant signaling axes.

These studies highlight complementary strategies for CAR-Treg-based immunotherapy in IBD while illustrating critical translational considerations. Antigen selection, tissue accessibility, lineage stability, and long-term persistence emerge as central determinants for therapeutic success. Moreover, they emphasize that although CAR-Treg systems offer unprecedented targeting precision, their functional behavior remains tightly coupled to the inflammatory and antigenic context of the intestinal microenvironment.

#### 3.3.2 Clinical CAR-T based approaches in IBD

Only one study has been published regarding the use of CAR-T therapy in IBD. Müller et al. reported a single-patient case in which a sustained remission of refractory ulcerative colitis was achieved following CD19-directed CAR-T therapy (91). The patient was a 21-year-old woman with severe multidrug-resistant ulcerative colitis that had previously failed numerous conventional and targeted therapies, including corticoids, aminosalycilates, anti-TNFα, anti-IL12/23p40, anti-integrin α4β7, anti-IL23p19, JAK inhibitors and sphingosine-1-phosphate modulators, none of which induced clinical remission. After the anti-CD19 CAR-T infusion, the patient achieved clinical and biochemical remission, which was maintained over a 14-week follow-up period, accompanied by mucosal healing and significant weight gain. The treatment was well-tolerated, with only an isolated grade 1 cytokine release syndrome and neutropenia (91). While this report provides an interesting proof-of-concept observation, it represents a single clinical case and should therefore be interpreted with considerable caution. At present, no additional preclinical or clinical studies support CD19 as a therapeutic target in IBD, and no conclusions regarding the efficacy or generalizability of CD19-directed CAR-T therapy can be drawn. Further mechanistic studies will be required to determine whether this observation reflects a true therapeutic effect or an isolated clinical finding.

Unlike several systemic autoimmune diseases, where autoreactive B cells play a central pathogenic role and B-cell depletion has demonstrated substantial clinical benefit, the contribution of B cells to IBD pathogenesis appears to be considerably more complex and less dominant (92, 93). Consistent with this concept, conventional B-cell-depleting strategies, such as anti-CD20 monoclonal antibodies, have not demonstrated meaningful clinical efficacy in IBD and have therefore not become part of the therapeutic armamentarium (94). Consequently, there is currently no compelling mechanistic or experimental evidence supporting CD19 as a disease-driving target in IBD. The rationale for CD19-directed CAR-T therapy in this setting therefore remains speculative and should not be extrapolated from its established efficacy in B-cell-mediated autoimmune diseases.

## 4 Conclusions

This systematic review provides a structured overview of the emerging field of CAR-T and Treg–based therapies in IBD, highlighting a rapidly evolving area characterized by strong mechanistic rationale but still limited clinical evidence.

A key concept emerging from the available data is the transition from broad immunosuppression toward precision immune modulation. While current therapies mainly target downstream inflammatory pathways, cellular immunotherapies aim to restore immune tolerance by directly reshaping dysregulated immune networks. In this context, Treg-based approaches offer a biologically grounded strategy to re-establish immune homeostasis, while CAR-engineered approaches introduce antigen-specific targeting, enabling more localized and controlled immunoregulation.

Preclinical studies included in this review provide important mechanistic insights into the potential and limitations of these new approaches in IBD. Treg-based therapies consistently demonstrate the capacity to modulate intestinal inflammation, but their effects appear to be highly context-dependent; factors such as cytokine environment, Treg stability, and cellular plasticity critically influence therapeutic outcomes, as illustrated by studies showing both anti-inflammatory effects and paradoxical pro-inflammatory skewing under specific conditions (55, 57, 58). Similarly, CAR-Treg approaches introduce an additional layer of specificity, with preclinical data supporting the feasibility of targeting both host-derived and microbiota-associated antigens. However, these studies also highlight key translational challenges, including limited *in vivo* persistence, antigen heterogeneity, and the complexity of the intestinal immune niche.

Clinical evidence remains even more limited. Treg-based therapies in IBD have been explored primarily in early-phase studies and case reports, demonstrating feasibility, acceptable safety profiles, and preliminary signals of biological and clinical activity. Importantly, these studies underscore critical aspects for future development, including the selection of stable Treg subsets, optimization of manufacturing protocols, and the need to ensure functional persistence in inflammatory environments. In contrast, clinical data on CAR-T approaches in IBD are currently restricted to isolated reports, precluding any definitive conclusions regarding efficacy or generalizability. Nevertheless, the remarkable success of CAR-T therapies in hematological malignancies, together with recent encouraging results in autoimmune diseases such as SLE, supports the broader concept of immune resetting through targeted cellular interventions.

Despite these limitations, several important implications for future research and clinical translation can be identified. A critical priority will be the identification of optimal antigen targets for CAR-based approaches in IBD, particularly given the complexity and heterogeneity of intestinal inflammation. In parallel, improving Treg stability in inflammatory environments will be essential to ensure sustained therapeutic effects.

Despite the considerable promise of adoptive Treg and CAR-Treg therapies, several important challenges remain before their incorporation into routine clinical practice. One of the principal limitations is the long-term persistence and phenotypic stability of transferred regulatory cells within the highly inflammatory intestinal microenvironment, where exposure to pro-inflammatory cytokines may impair suppressive function or promote cellular plasticity. The identification of suitable target antigens also remains particularly challenging in IBD owing to the marked heterogeneity of disease location, immune pathways, and microbial composition among patients. Manufacturing represents an additional translational hurdle, as the generation of stable, highly purified, GMP-compliant Treg products remains labor-intensive and costly. Furthermore, although early clinical studies have demonstrated encouraging safety profiles, long-term safety data are still lacking, particularly regarding durable immune modulation, unintended systemic immunosuppression, and potential off-target effects following genetic engineering. Importantly, different cellular strategies may partially overcome some of these limitations. Antigen-specific Tregs and CAR-Tregs are expected to improve tissue selectivity and reduce systemic immunosuppression compared with polyclonal Tregs, while advances in CAR engineering, gene editing, and synthetic biology may enhance lineage stability, persistence, and functional control. Advances in cell engineering, including next-generation CAR designs and gene-editing technologies, may facilitate the development of more precise and controllable therapeutic approaches. Furthermore, combinatorial strategies integrating cellular therapies with existing biologics or cytokine-targeting approaches may help overcome context-dependent limitations observed in preclinical models. From a clinical perspective, biomarker-driven approaches may be particularly relevant to identify patients most likely to benefit from these highly specialized therapies. Nevertheless, these approaches remain largely preclinical, and robust randomized clinical trials will ultimately be required to determine whether these theoretical advantages translate into meaningful clinical benefit for patients with IBD.

In conclusion, Treg-based cellular therapies, particularly CAR-Tregs, represent a promising new frontier in IBD treatment, offering the potential to move beyond conventional immunosuppression toward targeted and durable immune reprogramming. While current evidence remains limited, the convergence of advances in immunology, bioengineering, and translational medicine positions these approaches as key candidates for next-generation therapeutic strategies in IBD. Continued integration of mechanistic insights with clinical development will be essential to fully realize their therapeutic potential.

## Data Availability

The original contributions presented in the study are included in the article/supplementary material. Further inquiries can be directed to the corresponding authors.
